# Advancements in the fight against globally distributed OXA-48 carbapenemase: evaluating the new generation of carbapenemase inhibitors

**DOI:** 10.1128/aac.01614-24

**Published:** 2025-01-10

**Authors:** Michelle Outeda-García, Jorge Arca-Suárez, Emilio Lence, Arianna Rodriguez-Coello, Romina Maceiras, Tania Blanco-Martin, Paula Guijarro-Sánchez, Lucia Gonzalez-Pinto, Isaac Alonso-Garcia, Andrea García-Pose, Andrea Muras, Salud Rodriguez-Pallares, Cristina Lasarte-Monterrubio, Concepción Gonzalez-Bello, Juan Carlos Vázquez-Ucha, German Bou, Alejandro Beceiro

**Affiliations:** 1 Microbiology department, A Coruna University Hospital (CHUAC), Institute of Biomedical Research of A Coruna (INIBIC) 16811 , A Coruna, Spain; 2 CIBER de Enfermedades Infecciosas (CIBERINFEC), A Coruna, Spain; 3 Centro Singular de Investigación en Química Biolóxica e Materiais Moleculares (CiQUS), Departamento de Química Orgánica, Universidade de Santiago de Compostela, Jenaro de la Fuente s/n 16780 , Santiago de Compostela, Spain; Universita degli studi di roma La Sapienza, Rome, Italy

**Keywords:** OXA-48, carbapenem resistance, β-lactamase inhibitors, Enterobacterales, 1,6-diazabicyclo[3,2,1]octanes, bicyclic boronates

## Abstract

Carbapenemase OXA-48 and its variants pose a serious threat to the development of effective treatments for bacterial infections. OXA-48-producing Enterobacterales are the most prevalent carbapenemase-producing bacteria in large parts of the world. Although these bacteria exhibit low-level carbapenem resistance *in vitro*, the infections they cause are challenging to treat with conventional therapies, owing to their spread and complex detection in clinical settings. However, numerous β-lactamase inhibitors (BLIs) are currently in the pipeline or late clinical stages. To assess the potential of these compounds, this study compared the efficacy against OXA-48 of novel β-lactamase inhibitors, specifically the 1,6-diazabicyclo[3,2,1]octanes (DBOs) avibactam, relebactam, zidebactam, nacubactam, and durlobactam, along with the cyclic and bicyclic boronates vaborbactam, taniborbactam, and xeruborbactam. The extensive kinetics assays identified xeruborbactam, taniborbactam, and durlobactam, together with the already established avibactam, as BLIs with superior biochemical performance. Susceptibility testing further validated these findings but also demonstrated significantly improved bacterial killing by the DBOs zidebactam, nacubactam, and durlobactam. On the other hand, binding studies demonstrated the superior inhibitory capacity of the BLIs durlobactam and xeruborbactam. Combinations, such as cefepime/zidebactam, meropenem/nacubactam, and sulbactam/durlobactam, show promising activity against OXA-48-producing Enterobacterales, while ceftazidime/avibactam, cefepime/taniborbactam, and meropenem/xeruborbactam combinations also appear highly active, largely due to the excellent kinetics of these new inhibitors. Overall, this comprehensive analysis provides important insights into the effectiveness of new BLIs against OXA-48-producing Enterobacterales, highlighting xeruborbactam, durlobactam, and avibactam as leading candidates. Additionally, BLIs like zidebactam, nacubactam, and taniborbactam also showed potential in addressing the clinical challenges posed by OXA-48-mediated antimicrobial resistance.

## INTRODUCTION

Carbapenemases, particularly OXA-48 and its related OXA-48-like enzymes, pose a serious threat to health worldwide by undermining last-line antimicrobial therapies. Since 2010, OXA-48-producing Enterobacterales have spread globally, causing nosocomial outbreaks and regional endemicity and are now the most prevalent carbapenemase producers among Enterobacterales in most European and Eastern Mediterranean countries (1). These strains are challenging to treat due to their carbapenem resistance, which is often of low level *in vitro*, making detection in clinical settings problematic due to the high incidence of false negatives in biochemical tests (1). Despite having no effect on extended-spectrum cephalosporins, *bla*_OXA-48_-carrying isolates often display varying resistance to ceftazidime and cefepime due to ESBL co-production and porin loss (2). This scenario raises critical questions about the efficacy of conventional treatments and highlights the urgent need for innovative strategies to address OXA-48-induced resistance, as therapeutic options remain very limited (2). Fortunately, the strong impact of antimicrobial resistance on health has led to the development of effective agents that have recently been licensed (e.g., ceftazidime/avibactam and cefiderocol) or are undergoing development (e.g., novel β-lactam/β-lactam inhibitor combinations).

**Fig 1:**
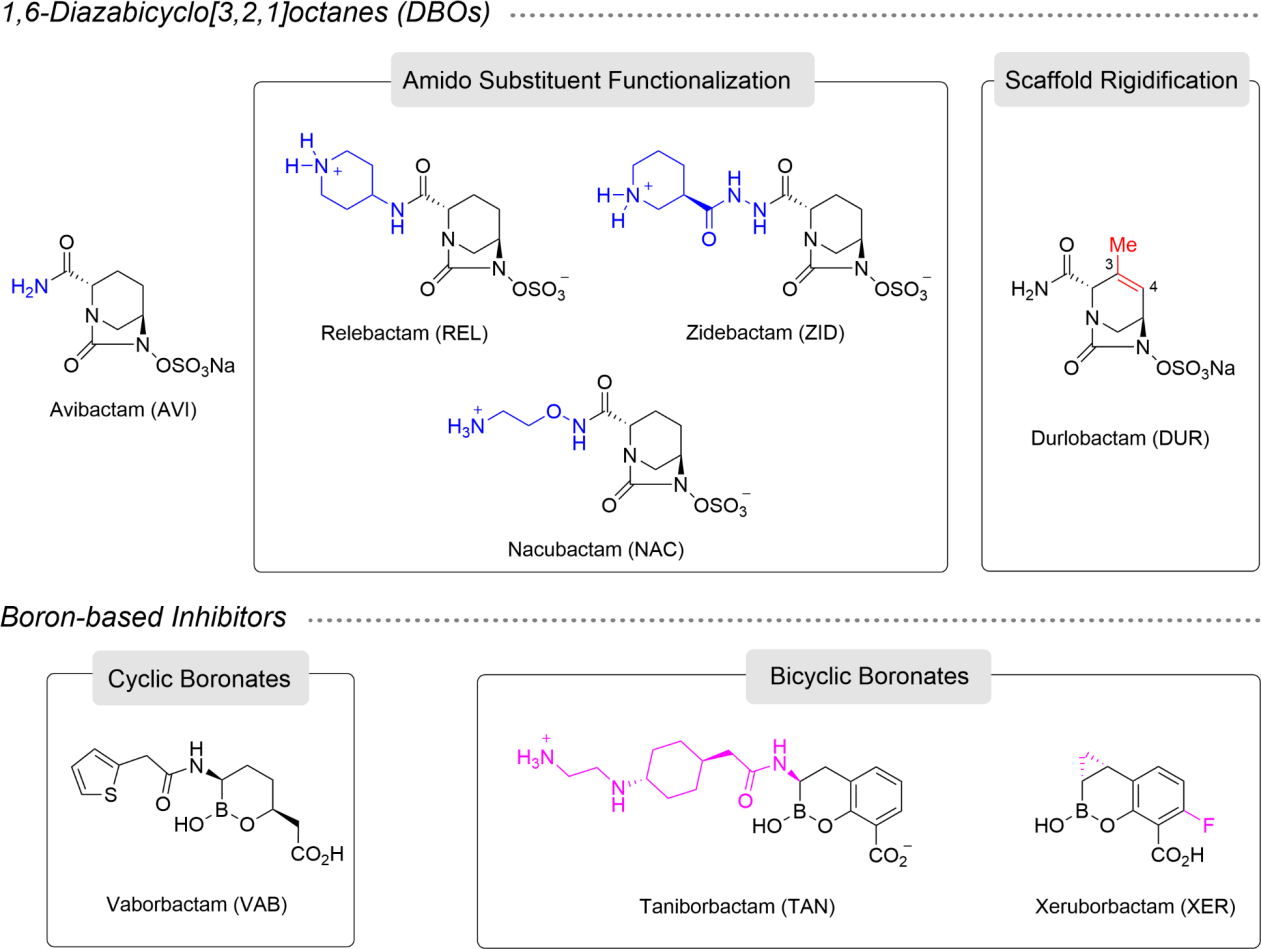
Chemical structures of the DBOs and boron-based β-lactamase inhibitors evaluated against OXA-48. The structural differences between avibactam and its derivatives involving the amido substituent (relebactam, zidebactam, and nacubactam) and its six-membered ring (durlobactam) are highlighted in blue and red, respectively. Among the boron-based inhibitors, note how xeruborbactam is a more rigid compound than taniborbactam because of the lack of a flexible side chain and the presence of a cyclopropyl moiety, both shown in purple.

Two main classes of new β-lactam inhibitors (BLIs) have recently been identified: (i) cyclic boronates (vaborbactam, the first boron-based BLI approved by the FDA) and bicyclic boronates (taniborbactam and xeruborbactam); and (ii) 1,6-diazabicyclo[3,2,1]octane (DBO) analogs, such as the FDA-approved inhibitors avibactam, relebactam, and durlobactam, and also zidebactam and nacubactam, which are undergoing clinical studies (Fig. 1) (3). Taniborbactam and xeruborbactam, which are under clinical development in combination with respectively cefepime and meropenem, cefiderocol or ceftibuten, represent a huge step forward in the BLI drug discovery field because of their ability to inhibit serine and metallo-β-lactamases. Thus, both compounds inhibit most class B β-lactamases in the nanomolar range and are also active against A, C, and some D class β-lactamases, therefore showing good potential for treating bacterial pathogens capable of simultaneously producing diverse β-lactamases (4, 5). Among the DBO analog, zidebactam has the ability to enhance the activity of cefepime against ESBL and carbapenemase producers by synergistically improving bacterial killing, attributed to its intrinsic anti-PBP2 activity (6). Furthermore, nacubactam enhances the efficacy of meropenem against class A ESBLs and carbapenemase-producing strains (7), while durlobactam, another DBO that exhibits broad-spectrum activity against class A, C, and D ß-lactamases, also shows affinity for PBP2 of Enterobacterales (8). Most of these combinations, except for sulbactam/durlobactam, have been shown to have potent activity against infections caused by carbapenemase-producing Enterobacterales, although some also display activity against other pathogens, e.g., cefepime in combination with taniborbactam or zidebactam against *Pseudomonas aeruginosa* (9, 10) or xeruborbactam in combination with different β-lactams against carbapenem-resistant *Acinetobacter baumannii* and *P. aeruginosa* (11, 12).

Given the clinical challenges posed by OXA-48, assessing the efficacy of new BLIs is crucial for determining optimal treatment strategies and developing new therapies targeting this enzyme. Information on the inhibition of this enzyme is currently limited, and the existing data have not been collectively evaluated or compared exhaustively. Therefore, it is not yet fully known which compounds most effectively block the enzyme. Additionally, the molecular basis of the different levels of efficacy of the BLIs against OXA-48 has not been analyzed in depth. To address these gaps, we herein present a detailed comparative analysis of the kinetic inhibition properties of the BLIs most recently developed against the OXA-48 carbapenemase. We further validated the kinetic findings by *in vitro* susceptibility testing using (i) an isogenic panel of recombinant strains expressing OXA-48 under varying permeability conditions and ii) a panel from a nationwide collection of genetically diverse clinical Enterobacterales isolates producing OXA-48. Finally, docking and molecular dynamics studies were used to provide insight into the molecular basis responsible for the inhibitory efficacy displayed by the most promising BLIs. Through these microbiological, biochemical, genetic, and structural studies, we aimed to enhance the understanding of OXA-48-related antimicrobial resistance, accurately assess the efficacy of new BLIs, and explore innovative therapeutic approaches for β-lactam-resistant infections.

## MATERIALS AND METHODS

### Bacterial strains and plasmids

*Escherichia coli* TG1 and *E. coli* HB4 (which shows inactivation of the major outer membrane porins OmpF/OmpC) were used as receptors to clone *bla*_OXA-48_ and later to evaluate the efficacy of novel BLIs. The *E. coli* HB4 strain was used to gain a more complete perspective, by evaluating the involvement in resistance of a low permeability background with the carbapenemase OXA-48, as well as coproduction of an ESBL and the carbapenemase; thus, the ESBL CTX-M-14 and OXA-48 were cloned together and separately into *E. coli* HB4. The characteristics of these strains and transformants are summarized in Table S1, and the cloning procedures are outlined in the supplemental methods.

The efficacy of these new BLIs was also evaluated against 18 genetically diverse WGS-characterized collection of OXA-48-positive Enterobacterales recovered as part of the Carbapenem-resistant Enterobacterales Genome Database Project (https://genomes.cnag.cat/incredble) listed in Table S2 (13). Combinations of clinical isolates (five pairs of *Klebsiella pneumoniae*, two pairs of *Enterobacter cloacae*, and two pairs of *E. coli*) were chosen for analysis: (i) nine strains carrying ESBLs; and (ii) nine strains (genetically closer available strains) without ESBLs.

### Kinetics for analysis of inhibition

OXA-48 was cloned, produced, and purified following the procedures described in the supplemental methods. The steady-state inhibition constants (*K_i_*_app_) and kinetics of inhibition (such as *k_2_/K* , *k*_off_, *K*_d_, and *t*_n_) were determined following previously established protocols, are also outlined in the supplemental methods.

### Antimicrobial susceptibility testing

To evaluate the efficacy of these novel BLIs against OXA-48-producers, the minimum inhibitory concentrations (MICs) of BL/BLI combinations that have recently been approved or are under development were determined, i.e., ceftazidime/avibactam, imipenem/relebactam, sulbactam/durlobactam, cefepime/zidebactam, cefepime/taniborbactam, meropenem/vaborbactam, meropenem/nacubactam, and meropenem/xeruborbactam. The MICs of each of the individual compounds (antibiotics or inhibitors) were also determined. The MICs were determined for the *E. coli* isogenic expressing OXA-48 (and both OXA-48 and CTX-M-14) to assess their activity against the carbapenemase; MICs were also determined for the 18 Enterobacterales clinical isolates in order to evaluate the new inhibitors against also their resistance concomitant mechanisms. The MICs were determined in triplicate experiments, and the EUCAST 13.0 clinical breakpoints were followed (http://www.eucast.org/clinical_breakpoints). The concentrations of BLIs were those recommended in the *in vitro* assays conducted to date, i.e., avibactam, relebactam, taniborbactam, and durlobactam were used at 4 mg/L, vaborbactam and xeruborbactam at 8 mg/L, and zidebactam and nacubactam in a 1:1 proportion with the partners.

For comparative evaluation of the activity of each inhibitor alone against the carbapenemase OXA-48, all inhibitors (at 4 mg/L) were tested in the presence of a carbapenem (ertapenem, adequate substrate) and a cephalosporin (ceftazidime, poor substrate) as reporter substrates. This prevented any possible masking effect produced by the different partner antibiotics. These assays were also performed with isogenic transformants and clinical isolates.

### Docking and molecular dynamics simulation studies

These studies were performed with the GOLD 2021.3.0 (14) program and using the protein coordinates from the crystal structures of OXA-48 from *K. pneumoniae* complexed with avibactam (PDB ID 4S2N [chain A]) (15). The ligand preparation (docking) used was similar to that previously described by our team (16).

The ligand minimization, generation, and minimization of OXA-48 inhibitor binary complexes and the molecular dynamics simulation of the resulting minimized complexes (40 ns) were carried following a previously described protocol (16). The enzyme structures were analyzed and represented using the programs PyMOL (17) and ChimeraX (18). The cpptraj module in Amber20 was used to analyze the trajectories and to calculate the root-mean-square deviation (rmsd) of the enzyme backbone (Cα, C, N, and O atoms) and the inhibitors during the simulation (19).

## RESULTS AND DISCUSSION

### Kinetics and inhibition studies

Nitrocefin hydrolysis rates for OXA-48 were *K*_m_ 56.5 µM (which allowed us to use nitrocefin at 300 µM as a reporter substrate in the studies of inhibition kinetics), and the catalytic constant (*k*_cat_) was 171.4 s^−1^. Thus, the specificity constant (*k*_cat_/*K*_m_) was 3.05 µM^−1^ s^−1^. These steady-state kinetic parameters for the substrate are in accordance with those previously obtained, providing good evidence of the viability of the purified OXA-48 enzyme (20).

#### The BLIs xeruborbactam, taniborbactam, and durlobactam displayed the most potent inhibitory activity against OXA-48

The kinetic data of the new BLIs against OXA-48 are summarized in Table 1. The bicyclic boronates, xeruborbactam and taniborbactam, and the DBO durlobactam exhibited the strongest inhibition kinetics, with lower *K_i_*_app_ values, indicating high potency in inhibiting the enzyme activity in the presence of the substrate. Thus, the lowest *K_i_*_app_ against OXA-48 were those of xeruborbactam, taniborbactam, and durlobactam obtained in the nM range (*K_i_*_app_ of 0.018, 0.075, and 0.103 µM, respectively), followed by avibactam (3.8 µM). Relebactam, zidebactam, and nacubactam displayed a *K_i_*_app_ in the μM range (>800 µM), which, however, was sufficient to produce limited inhibition of β-lactamases in this study (but still far from the inhibition results obtained for xeruborbactam, taniborbactam, or durlobactam). Meanwhile, vaborbactam displayed intermediate *K_i_*_app_ values (43.6 µM). Thus, the lower *K_i_*_app_ values highlight the high potency of xeruborbactam, taniborbactam and durlobactam.

**TABLE 1:** Kinetics parameters of OXA-48 with β-lactamase inhibitors^*a*^

β-Lactamase inhibitor		*K*_i app_ (µM)	*k_2_/K* (M^−1^ s^−1^)	*t*_n_	*k*_off_ (s^−1^)	*t_1/2_* (min)	*K_d_* (nM)
DBOs	Avibactam	3.815 ± 1.334	822.74 ± 182.83	2	5.5 × 10^−6^ ± 0.7 x10^−6^	2117.5 ± 272.2	6.68
	Relebactam	>800	ND	350	9.5 × 10^−5^ ± 0.7 x10^−5^	121.9 ± 9.1	ND
	Zidebactam	>800	20.11 ± 7.75	50	3 × 10^−5^ ± 0.6 x10^−5^	385.1 ± 74.3	1.37 × 10^3^
	Nacubactam	>800	4.88 ± 2.11	70	2.0 × 10^−4^ ± 0.2 x10^−4^	57.7 ± 7.5	40.98 × 10^3^
	Durlobactam	0.103 ± 0.024	1.42 × 10^5^ ± 0.38 x10^5^	1	9.5 × 10^−6^ ± 0.7 x10^−6^	1215.8 ± 212.8	0.07 ± 0.01
Boronates	Vaborbactam	43.624 ± 10.641	ND	>3,000	2.6 × 10^−4^ ± .5 10^−4^	44.4 ± 1.6	ND
	Taniborbactam	0.075 ± 0.015	ND	200	2.3 × 10^−4^ ± 0.3 x10^−4^	50.2 ± 8.2	ND
	Xeruborbactam	0.018 ± 0.002	3.13 × 10^5^ ± 0.63 x10^5^	1	2.0 × 10^−4^ ± 0.3 x10^−4^	57.7 ± 9.6	0.63

^*a*^ Data represent the means of three independent experiments. ND, not available for determination.

However, *K_i_*_app_ reflects the effectiveness of inhibition in the context of enzyme activity rather than direct binding affinity, which is better represented by *K_d_*. Thus, equilibrium dissociation constants (*K_d_*) were also measured and found to be in the nanomolar range, particularly for durlobactam and, to a lesser extent, for xeruborbactam and avibactam. By contrast, zidebactam and nacubactam yielded high *K_d_* values primarily due to low *k_2_/K* (association rate) values, suggesting weaker binding affinity in the resting state of OXA-48.

These findings are in accordance with those recently published by Lomovskaya et al., who evaluated the *K_i_*_app_ of several inhibitors and found that durlobactam, xeruborbactam, and taniborbactam displayed the strongest inhibitory activity against OXA-48 (21). In general, the data are also consistent with the findings of studies that have evaluated the kinetics of some of these inhibitors individually against OXA-48, such as the *K_i_* to avibactam, relebactam, xeruborbactam (22, 23), and taniborbactam (24). Xeruborbactam yielded the highest inhibitory potency, as observed in the studies published to date with this compound. Thus, our findings are consistent with those of a study by Hecker et al. showing that xeruborbactam has higher inhibitory activity (*K_i_*) for OXA-48 than avibactam (96-fold) or vaborbactam (50,000-fold) (5).

#### Xeruborbactam, durlobactam, and avibactam also displayed high efficiency of inhibition

The highest values of inhibition efficiency, measured as *k_2_/K*, were observed for xeruborbactam and durlobactam, which were 3.1 × 10^5^ and 1.4 × 10^5^ M^−1^s^−1^, respectively, significantly higher than for avibactam, 822.7 M^−1^s^−1^. A *k_2_*/*K* value for avibactam of 1.4 × 10^3^ M^−1^s^−1^ has previously been reported by Ehmann et al. (25), similar to the value obtained in our study. The very high inactivation efficiency of xeruborbactam was previously demonstrated by Tsivkovski et al. (23). The *k_2_*/*K* values could not be obtained for relebactam, taniborbactam, or vaborbactam because the *K*_obs_ constants could not be determined under the experimental conditions.

Finally, the partition ratio *t*_n_ values were consistent with the other kinetics data (a low partition ratio indicates high inhibitory efficiency, as fewer substrate and inhibitor molecules are hydrolyzed before β-lactamase inactivation, whereas a high ratio suggests lower efficiency). The data showed a significantly lower I : E ratio for xeruborbactam (*t*_n_  =  1), durlobactam (*t*_n_  =  1), and avibactam (*t*_n_  =  2) than for the other inhibitors tested (*t*_n_  =  50–> 3,000), indicating 90% inhibition of OXA-48 at a much lower concentration.

#### Durlobactam and avibactam yielded the slowest dissociation of the carbapenemase

Separate experiments (*k*_off_) indicated that the compounds with the lowest off-rate (dissociation rate constant) were durlobactam and avibactam (9.5 × 10^−6^ and 5.5 × 10^−6^ s^−1^, respectively), resulting in a residence time (*t_1/2_*) of 1215.8 and 2117.5 min, respectively, indicating very slow recovery of carbapenemase activity. The residence times of relebactam and zidebactam were intermediate (121.9 and 385.1 min, respectively), while those of taniborbactam, vaborbactam, nacubactam, and xeruborbactam were shorter (44.4–57.7 min). Lower *k*_off_ values indicate a longer half-life between inhibitor and enzyme, and avibactam and durlobactam inhibition thus displayed a very slow reversible mechanism. Our results with avibactam (studied in more detail than other BLIs) are consistent with those previously published by Lahiri et al. (26) (*k*_off_ of 1.2 × 10^−5^ s^−1^ and *t*_1/2_ of 1,000 min) and Ehmann et al. (25) (*k*_off_ of 1.2 × 10^−5^ s^−1^). Likewise, the *k*_off_ of durlobactam obtained in this study was also consistent with that reported by Shapiro and Gao (27) (2.5 × 10^−5^ s^−1^).

Altogether, our findings reveal new kinetic data on OXA-48 inhibition, showing potential for new BL/BLI combinations against this carbapenemase. Xeruborbactam and durlobactam displayed the highest inhibition for OXA-48, while durlobactam and avibactam showed the lowest off-rates. The other inhibitors evaluated in this and previous studies generally show poorer inhibition kinetics (4). Thus, the boronate xeruborbactam and DBOs durlobactam and avibactam displayed the best biochemical performance against the OXA-48 enzyme.

### Antimicrobial susceptibility assays

#### Antimicrobial activity of the BL/BLIs against OXA-48-producing transformants

##### BL/BLIS combinations in late stage of development

The MIC assays performed in *E. coli* transformants revealed that the effect of OXA-48 on the susceptibility to the tested β-lactam antibiotics largely depends on the permeability of the bacteria (Table 2). Thus, in *E. coli* TG1 with both OmpF/C porins functional, the presence of OXA-48 scarcely increased the MICs of carbapenems; however, when this enzyme was expressed in the porin-deficient background of *E. coli* HB4, resistance to carbapenems increased greatly, as expected. The co-production of OXA-48 and an ESBL, a very frequent phenomenon among multidrug-resistant Enterobacterales, was also evaluated. One of the most widespread ESBLs worldwide, CTX-M-14, was chosen for this purpose. As expected, the MICs of cephalosporins and sulbactam were particularly high for the *E. coli* HB4 CTX-M-14 strain, and therefore the *E. coli* HB4 producing both OXA-48 and CTX-M-14 yielded elevated MICs for all β-lactam antibiotics tested.

**TABLE 2:** Minimum inhibitory concentrations (mg/L) of novel combinations β-lactam/β-lactamase inhibitors in development against *E. coli* TG1 and HB4 (low permeability) expressing OXA-48 carbapenemase and CTX-M-14 ESBL

Strain	MIC of^*b*^:												
	CAZ	CAZ/AVI	IMP	IMP/REL	SUL	SUL/DUR	FEP	FEP/ZID	FEP/TAN	MER	MER/VAB	MER/NAC	MER/XER
*E. coli* TG1	0.25	≤0.06^*a*^	≤0.06	≤0.06	32	≤0.06^*a*^	≤0.06	≤0.06	≤0.06	≤0.06	≤0.06	≤0.06	≤0.06
*E. coli* TG1 (OXA-48)	0.25	≤0.06^*a*^	0.12	0.12	32	≤0.06^*a*^	≤0.06	≤0.06	≤0.06	≤0.06	≤0.06	≤0.06	≤0.06
*E. coli* HB4 Δ*ompC/F*	1	0.5	≤0.06	≤0.06	32	≤0.06^*a*^	1	≤0.06	0.5	0.12	≤0.06	≤0.06	≤0.06
*E. coli* HB4 Δ*ompC/F* (OXA-48)	1	0.5	16	16	64	≤0.06^*a*^	2	0.25	1	32	32	1	≤0.06
*E. coli* HB4 Δ*ompC/F* (CTX-M-14)	16	0.5	0.25	0.12	32	≤0.06^*a*^	16	1	1	0.5	0.25	0.12	≤0.06
*E. coli* HB4 Δ*ompC/F* (CTX-M-14 + OXA-48)	16	0.5	16	16	64	≤0.06^*a*^	16	1	1	32	32	1	≤0.06

^*a*^ No growth, the MICs to avibactam and durlobactam are lower than the used concentration (4 mg/L).

^*b*^ CAZ: ceftazidime; AVI: avibactam; IMP: imipenem; REL: relebactam; SUL: sulbactam; DUR: durlobactam; FEP: cefepime; ZID: zidebactam; TAN: taniborbactam; MER: meropenem; VAB: vaborbactam; NAC: nacubactam; XER: xeruborbactam.

Some DBOs, such as zidebactam, nacubactam, and durlobactam exhibit antimicrobial activity by themselves, i.e., they inhibit both β-lactamases and likely PBP2, thus displaying synergy as β-lactam “enhancers” (22, 28). This synergistic action should be taken into account when interpreting the results of microbiological assays. Thus, for TG1 and HB4 *E. coli*, the MICs of zidebactam, nacubactam, and durlobactam alone were 0.12–2 mg/L, whereas most other β-lactamase inhibitors yielded MICs ≥ 32 mg/L (Table 3), further supporting the previously mentioned findings. The implications of this are important; as in the case of zidebactam and nacubactam, the inhibitor is tested in a 1:1 ratio with the antimicrobial partner, while durlobactam is tested at a constant concentration of 4 mg/L for all MIC dilutions.

**TABLE 3:** Minimum inhibitory concentrations (mg/L) of individual β-lactamase inhibitors in *E. coli* transformants

Strain	MIC of^*a*^:							
	AVI	REL	ZID	NAC	DUR	VAB	TAN	XER
*E. coli* TG1	4	32	0.12	2	0.12	≥128	≥128	32
*E. coli* TG1 (OXA-48)	4	64	0.12	2	0.12	≥128	≥128	32
*E. coli* HB4 Δ*ompC/F*	8	≥128	0.5	2	0.5	≥128	≥128	16
*E. coli* HB4 Δ*ompC/F* (OXA-48)	8	≥128	0.5	2	1	≥128	≥128	16
*E. coli* HB4 Δ*ompC/F* (CTX-M-14)	16	≥128	0.5	2	1	≥128	≥128	16
*E. coli* HB4 Δ*ompC/F* (CTX-M-14 + OXA-48)	16	≥128	0.5	2	1	≥128	≥128	16

^*a*^ AVI: avibactam; REL: relebactam; ZID: zidebactam; NAC: nacubactam; DUR: durlobactam; VAB: vaborbactam; TAN: taniborbactam; XER: xeruborbactam.

Therefore, we found that the BL/BLI combinations in development that were most active against the OXA-48-producing *E. coli* TG1 and HB4 transformants were sulbactam/durlobactam and meropenem/xeruborbactam, which prevented bacterial growth (MIC ≤ 0.06 mg/L). *E. coli* HB4 producing OXA-48 was also very susceptible to meropenem/nacubactam. In the *E. coli* HB4 transformant producing both CTX-M-14 and OXA-48, avibactam, zidebactam, and taniborbactam enhanced the activity of the cephalosporin partners, although mainly due to inhibition of CTX-M-14 ESBL activity against these cephalosporins, as OXA-48 does not exhibit strong hydrolysis of these antibiotics. The addition of the BLIs to imipenem/relebactam and meropenem/vaborbactam combinations did not increase the activity against OXA-48-producing transformants (Table 2). Thus, the microbiological assays showed that zidebactam, nacubactam, and durlobactam acted as inhibitors but also displayed antimicrobial activity (Table 3), while xeruborbactam yielded excellent inhibition of OXA-48 and also of the class A β-lactamase CTX-M-14 in the *E. coli* HB4 transformant, as previously reported (5, 29).

##### BLIs in combination with ertapenem and ceftazidime

The OXA-48-producing transformants were subsequently challenged against the panel of new BLIs in combination with good (ertapenem) and poor (ceftazidime) OXA-48 substrates in order to identify the best candidate inhibitors.

All ertapenem/BLI combinations, except relebactam and vaborbactam, displayed significant activity against the OXA-48-producing *E. coli* hosts, decreasing the MICs of ertapenem with avibactam, taniborbactam, and xeruborbactam 8–256-fold, while zidebactam, nacubactam, and durlobactam totally inhibited bacterial growth. Xeruborbactam and avibactam were the strongest inhibitors of *E. coli* HB4 transformant coproducing CTX-M-14 and OXA-48 (decreasing the ertapenem MIC by 256-fold), followed by taniborbactam (which caused an 8-fold decrease in the MIC) (Table 4).

**TABLE 4:** Minimum inhibitory concentrations (mg/L) of ertapenem in combination with novel β-lactamase inhibitors (4 mg/L) against *E. coli* TG1 and *E. coli* HB4 (low permeability) expressing OXA-48 carbapenemase OXA-48 carbapenemase and CTX-M-14 ESBL

Strain	MIC of^*b*^:								
	ERT	ERT/AVI	ERT/REL	ERT/ZID	ERT/NAC	ERT/DUR	ERT/VAB	ERT/TAN	ERT/XER
*E. coli* TG1	≤0.06	≤0.06^*a*^	≤0.06	≤0.06^*a*^	≤0.06^*a*^	≤0.06^*a*^	≤0.06	≤0.06	≤0.06
*E. coli* TG1 (OXA-48)	1	≤0.06^*a*^	0.5	≤0.06^*a*^	≤0.06^*a*^	≤0.06^*a*^	1	≤0.06	≤0.06
*E. coli* HB4 Δ*ompC/F*	0.12	≤0.06	≤0.06	≤0.06^*a*^	≤0.06^*a*^	≤0.06^*a*^	≤0.06	≤0.06	≤0.06
*E. coli* HB4 Δ*ompC/F* (OXA-48)	32	0.12	16	≤0.06^*a*^	≤0.06^*a*^	≤0.06^*a*^	32	4	0.12
*E. coli* HB4 Δ*ompC/F* (CTX-M-14)	0.5	0.12	0.25	≤0.06^*a*^	≤0.06^*a*^	≤0.06^*a*^	0.5	0.12	0.12
*E. coli* HB4 Δ*ompC/F* (CTX-M-14 + OXA-48)	32	0.12	32	≤0.06^*a*^	≤0.06^*a*^	≤0.06^*a*^	32	4	0.12

^*a*^ No growth, the MICs to avibactam, zidebactam, nacubactam and durlobactam are lower than the used concentration (4 mg/L).

^*b*^ ERT: ertapenem; AVI: avibactam; REL: relebactam; ZID: zidebactam; NAC: nacubactam; DUR: durlobactam; TAN: taniborbactam; VAB: vaborbactam; XER: xeruborbactam.

In accordance with these findings, when the *E. coli* transformants were tested against ceftazidime (scarcely hydrolyzed by OXA-48) in combination with the BLIs, minimal effects were observed; however, in the transformants of *E. coli* HB4 producing CTX-M-14, an increase in the ceftazidime MIC was observed. Zidebactam, nacubactam and durlobactam produced the best results, possibly due to their synergistic antimicrobial activity and inhibition potency against OXA-48/CTX-M-14 (Table S3). Avibactam in combination with ceftazidime, followed by taniborbactam and xeruborbactam, also showed high activity against the *E. coli* HB4 transformant producing CTX-M-14 and also against the *E. coli* HB4 transformant coproducing CTX-M-14 and OXA-48.

### Antimicrobial activity of the BL/BLIs against Enterobacterales clinical isolates

#### BL/BLIS combinations in late stage of development

Regarding the collection of clinical strains of OXA-48-producing Enterobacterales, a study of the sequences of the major porins *ompk35*, *ompK36*, *ompK37*, and *ompA* from *K. pneumoniae* (and homolog porins in *E. coli* and *E. cloacae*) was conducted following the methodology described in the supplemental material. The porins were found to be intact, and no changes that led to loss of function or a decrease of permeability in any of the clinical isolates were detected. As reflected in Table S4, zidebactam, nacubactam and durlobactam each displayed antimicrobial activity, although not with all strains, in a broad range of 0.25–≥128, 1–≥128, and 0.25–4 mg/L, respectively. To a lesser extent, xeruborbactam also shows certain activity, with MICs ranging from 32 to ≥128 mg/L. This was reflected in the MICs for the BLIs with antibiotics. Thus, as shown in Table 5, the ceftazidime/avibactam combination showed excellent activity, which was particularly evidenced in ESBL-producing strains. The synergistic effect of the combinations of zidebactam and taniborbactam with cefepime also proved to be excellent, reducing cefepime MICs 2–512-fold, which is consistent with previous findings (30). Regarding combinations with carbapenems, neither imipenem/relebactam nor meropenem/vaborbactam showed any improvement compared with carbapenems alone, as expected from the hydrolytic profile of the antibiotic and the inhibition spectrum of these BLIs. By contrast, while nacubactam had a minimal impact on decreasing meropenem MIC (0–32-fold), xeruborbactam caused a significant reduction in meropenem MICs by completely inhibiting bacterial growth in most of the isolates due mainly to their excellent β-lactamase inhibition kinetics and a slight antimicrobial activity. The MICs of sulbactam/durlobactam were the same (total growth inhibition, MIC ≤ 0.06 mg/L) with all strains, except one (MIC 1 mg/L). This is because durlobactam was tested at 4 mg/L (which is the proposed concentration for clinical methodology), again highlighting the intrinsic antibacterial activity of durlobactam against all isolates (Table S4), as sulbactam was not at all effective against all the tested isolates (MIC ≥ 64 mg/L, Table 5). Sulbactam/durlobactam is a novel combination specifically designed for carbapenem-resistant *A. baumannii* isolates (8); however, it displays some activity against Enterobacterales, as shown in this study and others, with a significant percentage of strains yielding a MIC > 4 mg/L for durlobactam (31). Therefore, the combination of durlobactam with an antibiotic, such as cefepime, might be a promising treatment option against OXA-48-producing Enterobacterales because of its excellent inhibition kinetics.

**TABLE 5:** Minimum inhibitory concentrations (mg/L) of novel combinations β-lactam/β-lactamase inhibitors in development against Enterobacterales clinical isolates carrying OXA-48, and with ESBLs (*n* = 10) or without ESBLs (*n* = 10)

Species		Strain no.	MLST	MIC of^*b*^:												
				CAZ	CAZ/AVI	IMP	IMP/REL	FEP	FEP/ZID	FEP/TAN	SUL	SUL/DUR	MER	MER/VAB	MER/NAC	MER/XER
*E. coli*	ESBL	177	131	128	0.25	2	0.5	≥256	0.5	0.5	64	≤0.06^*a*^	0.5	0.5	0.5	≤0.06
		103	10	4	0.5	2	2	16	0.25	0.12	64	≤0.06^*a*^	0.25	0.25	0.25	≤0.06
	No ESBL	264	131	0.25	0.25	0.5	0.5	2	≤0.06	0.5	64	≤0.06^*a*^	1	1	0.5	≤0.06
		224	127	0.25	0.25	2	2	0.5	≤0.06	0.12	128	≤0.06^*a*^	0.5	0.5	0.5	≤0.06
*E. cloacae*	ESBL	272	78	≥256	1	2	2	16	1	0.25	128	≤0.06^*a*^	1	1	0.5	≤0.06
		235	171	128	1	≥256	≥256	≥256	4	4	≥256	≤0.06^*a*^	128	128	4	0.5
	No ESBL	281	78	0.5	0.25	8	8	1	0.25	0.12	128	≤0.06^*a*^	1	1	0.5	≤0.06
		231	171	1	0.12	2	2	1	≤0.06	0.5	64	≤0.06^*a*^	1	1	0.25	≤0.06
*K. pneumoniae*	ESBL	19	11	32	0.5	1	1	16	0.25	0.25	64	≤0.06^*a*^	1	1	0.5	≤0.06
		238	15	32	0.5	2	2	16	0.25	0.12	128	≤0.06^*a*^	1	1	0.5	≤0.06
		413	147	32	0.5	16	16	128	0.5	1	128	1	16	16	2	0.12
		23	392	128	0.5	4	2	32	0.5	0.12	128	≤0.06^*a*^	1	1	1	≤0.06
		125	307	64	0.5	4	2	16	0.12	0.5	64	≤0.06^*a*^	2	2	1	≤0.06
	No ESBL	497	11	0.5	0.25	4	2	0.5	0.12	0.25	128	≤0.06^*a*^	1	1	1	≤0.06
		71	15	0.5	0.5	2	2	0.5	0.12	0.12	128	≤0.06^*a*^	2	2	1	≤0.06
		237	147	0.25	0.25	2	1	0.5	0.12	0.12	64	≤0.06^*a*^	1	1	0.5	≤0.06
		194	392	0.5	0.5	2	1	0.25	≤0.06	0.12	64	≤0.06^*a*^	2	2	1	≤0.06
		170	307	0.5	0.25	4	2	0.25	0.12	≤0.06	64	≤0.06^*a*^	1	1	1	≤0.06

^*a*^ No growth, the MICs to durlobactam are lower than the used concentration (4 mg/L).

^*b*^ CAZ: ceftazidime; AVI: avibactam; IMP: imipenem; REL: relebactam; FEP: cefepime; ZID: zidebactam; TAN: taniborbactam; SUL: sulbactam; DUR: durlobactam; MER: meropenem; VAB: vaborbactam; NAC: nacubactam; XER: xeruborbactam.

Several studies have tested the MICs of these BL/BLI combinations against collections of Enterobacterales with similar findings; e.g., Karlowsky et al. reported MIC_50/90_ values of 0.5/2 mg/L for cefepime/zidebactam, among OXA-48-carrying Enterobacterales (32). Cefepime/taniborbactam has also been evaluated with several collections of Enterobacterales, showing high efficacy (MIC_50/90_ of 0.5/2 mg/L) against isolates carrying OXA-48 or KPC carbapenemases, improving ceftazidime/avibactam and ceftolozane/tazobactam (24). Even better results were obtained with the meropenem/xeruborbactam combination, which improved the MICs of cefepime/taniborbactam or ceftazidime/avibactam 16–32-fold (21). In general, the results obtained in our study are consistent with those of studies comparing some of these combinations.

#### BLIs in combination with ertapenem and ceftazidime

Table 6 shows the MICs of all BLIs in combination with ertapenem as partner. As observed above for the isogenic OXA-48 transformants, although relebactam and vaborbactam were not able to reduce the MIC to ertapenem against OXA-48-producing isolates, the other BLIs were able to drastically reduce the MIC 32–64-fold in most cases.

**TABLE 6:** Minimum inhibitory concentrations (mg/L) of ertapenem in combination with novel β-lactamase inhibitors (4 mg/L) against Enterobacterales clinical isolates carrying OXA-48, and with ESBLs (*n* = 10) or without ESBLs (*n* = 10)

Species		Strain no.	MLST	MIC of^*b*^:								
				ERT	ERT/AVI	ERT/REL	ERT/ZID	ERT/NAC	ERT/DUR	ERT/VAB	ERT/TAN	ERT/XER
*E. coli*	ESBL	177	131	4	≤0.06	4	≤0.06	0.5	≤0.06^*a*^	4	0.25	≤0.06
		103	10	4	≤0.06	4	≤0.06	≤0.06	≤0.06^*a*^	4	0.5	≤0.06
	No ESBL	264	131	4	≤0.06	4	≤0.06	≤0.06	≤0.06^*a*^	4	0.25	≤0.06
		224	127	2	≤0.06	2	≤0.06	≤0.06	≤0.06^*a*^	2	0.12	≤0.06
*E. cloacae*	ESBL	272	78	16	0.25	8	≤0.06	≤0.06	≤0.06^*a*^	8	0.5	0.5
		235	171	≥256	16	≥256	≤0.06	0.5	≤0.06^*a*^	≥256	32	8
	No ESBL	281	78	4	≤0.06	4	≤0.06	≤0.06	≤0.06^*a*^	4	0.25	≤0.06
		231	171	8	≤0.06	8	≤0.06	≤0.06	≤0.06^*a*^	8	0.25	≤0.06
*K. pneumoniae*	ESBL	19	11	4	≤0.06	4	0.12	0.25	≤0.06^*a*^	4	0.25	≤0.06
		238	15	4	≤0.06	4	≤0.06	0.25	≤0.06^*a*^	4	0.25	≤0.06
		413	147	≥256	4	≥256	8	8	≤0.06^*a*^	≥256	32	2
		23	392	4	≤0.06	4	≤0.06	≤0.06	≤0.06^*a*^	4	0.25	≤0.06
		125	307	8	0.12	8	0.25	0.25	≤0.06^*a*^	8	0.5	≤0.06
	No ESBL	497	11	8	0.12	8	0.12	≤0.06	≤0.06^*a*^	8	0.5	≤0.06
		71	15	8	0.12	8	≤0.06	0.25	≤0.06^*a*^	8	1	≤0.06
		237	147	4	≤0.06	4	≤0.06	≤0.06	≤0.06^*a*^	4	0.25	≤0.06
		194	392	4	≤0.06	4	≤0.06	≤0.06	≤0.06^*a*^	4	0.25	≤0.06
		170	307	4	1	4	≤0.06	0.5	≤0.06^*a*^	4	1	1

^*a*^ No growth, the MICs to durlobactam are lower than the used concentration (4 mg/L).

^*b*^ ERT: ertapenem; AVI: avibactam; REL: relebactam; ZID: zidebactam; NAC: nacubactam; DUR: durlobactam; VAB: vaborbactam; TAN: taniborbactam; XER: xeruborbactam.

Finally, regarding the BLIs combined with ceftazidime against clinical isolates, as expected, the presence of ESBL was essential to increase the MICs of this antibiotic (Table S5). We observed that all BLIs were able to decrease the MIC of ceftazidime against ESBL-producing strains by 8–512-fold. Zidebactam, nacubactam, and durlobactam were again able to inhibit bacterial growth of most of Enterobacterales isolates tested.

In general, the microbiological data are consistent with the biochemical data obtained with the kinetics performed with the purified OXA-48 enzyme. The findings highlight xeruborbactam and durlobactam, and to a lesser degree taniborbactam, for their excellent ability to inhibit OXA-48. Furthermore, mainly zidebactam, nacubactam, and durlobactam are also highlighted for their antimicrobial activity as well as their ability to inhibit carbapenemase OXA-48, with durlobactam particularly standing out for this dual activity.

### Binding studies

To provide an insight of the molecular basis responsible for the excellent inhibitory capacity of durlobactam, which proved to be the most potent DBO analog studied here, *in silico* studies were performed. To this end, the binding mode of durlobactam in the active site of OXA-48 was studied by docking and further validated by molecular dynamics (MD) simulations by using AMBER to provide a reliable OXA-48 durlobactam model (Michaelis–Menten), which triggers covalent modification (induced-fit model) (for details, see the supplemental methods). The same studies were also performed with avibactam and relebactam to compare the results.

**Fig 2:**
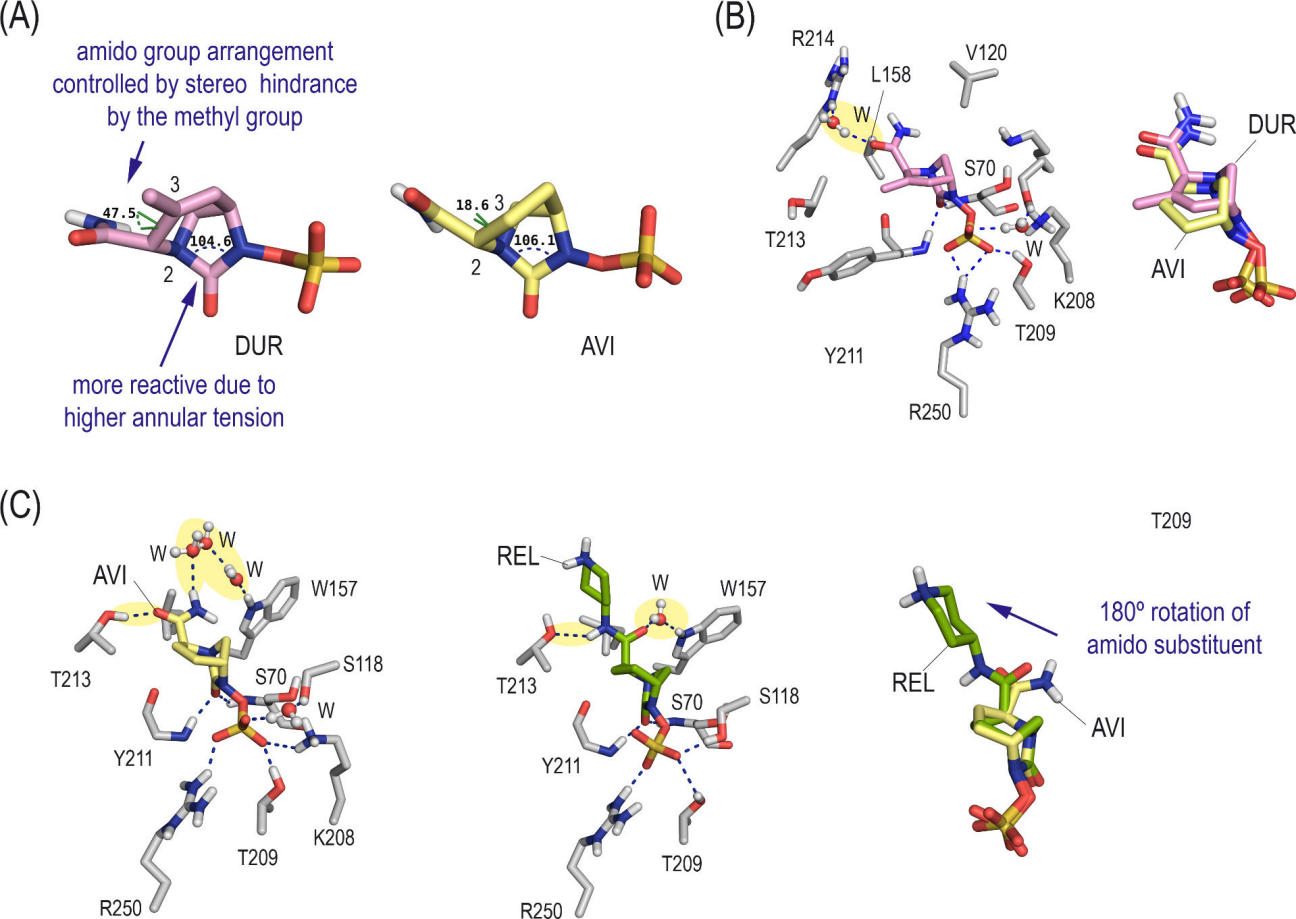
Comparison of the binding mode of DUR, AVI, and REL in OXA-48 active site (Michaelis complex), which triggers enzyme inactivation, obtained by MD simulations. (**A**) Comparison of the bond angles around the carbonyl carbon atom (urea moiety) and dihedral angle between the substituents in positions 2 and 3 in DUR and AVI. Note that DUR would be more reactive towards covalent modification because of the higher annular tension, and the position of its CONH_2_ moiety would be fixed by the presence of the methyl group in position 3. (**B**) Detailed view of the interactions with DUR in OXA-48 active site. A snapshot taken after 40 ns of simulation is shown. Superposition of DUR and AVI arrangements is also shown. Side chain residues are shown and labeled. Hydrogen-bonding interactions are shown as dashed blue lines. Relevant contacts of the CONH_2_ group with OXA-48 are shaded yellow. Note that the defined arrangement of the CONH_2_ moiety promotes additional contacts with R214, which are absent with AVI. (**C**) Main contacts of AVI and REL with the active site residues and superposition of their arrangements. Snapshots taken after 40 ns of simulation, respectively, are shown. Note how the introduction of the large substituent in the CONH_2_ moiety causes 180° rotation of this substituent, thus causing changes in the overall recognition of the inhibitor.

First, the comparison of the minimized 3D structures of durlobactam and avibactam clearly showed that the inclusion of a double bond between positions 3 and 4 in avibactam induced an important increase in the annular tension of the urea moiety, which causes the covalent modification of the catalytic serine (Fig. 2A). In addition, the stereo hindrance induced by the presence of the methyl group in the scaffold would also maximize the effectiveness of the interactions of the CONH_2_ group, which proved to be key in binding and activity. In addition, our MD simulations studies showed that the overall arrangement of durlobactam and avibactam would be similar and stable during simulation (Fig. S1); durlobactam would promote additional interactions with OXA-48, which were not observed for avibactam (Fig. 2B; Fig. S2). Specifically, a strong hydrogen-bonding interaction of the CONH_2_ group with the guanidinium group in R214 via a water molecule and an apolar interaction with T213 was identified. These favorable contacts along with the higher reactivity against nucleophilic attack may explain the higher inhibitory capacity of durlobactam. On the other hand, the introduction of substituents in the CONH_2_ group of avibactam, such as those observed in relebactam, would cause a 180° turn of this group to accommodate the bulky substituent in the active site (Fig. 2C). As a result, the urea moiety in relebactam would be located further away and with less optimal arrangement to undergo a nucleophilic attack by the catalytic serine residue, which may explain its poor activity.

**Fig 3:**
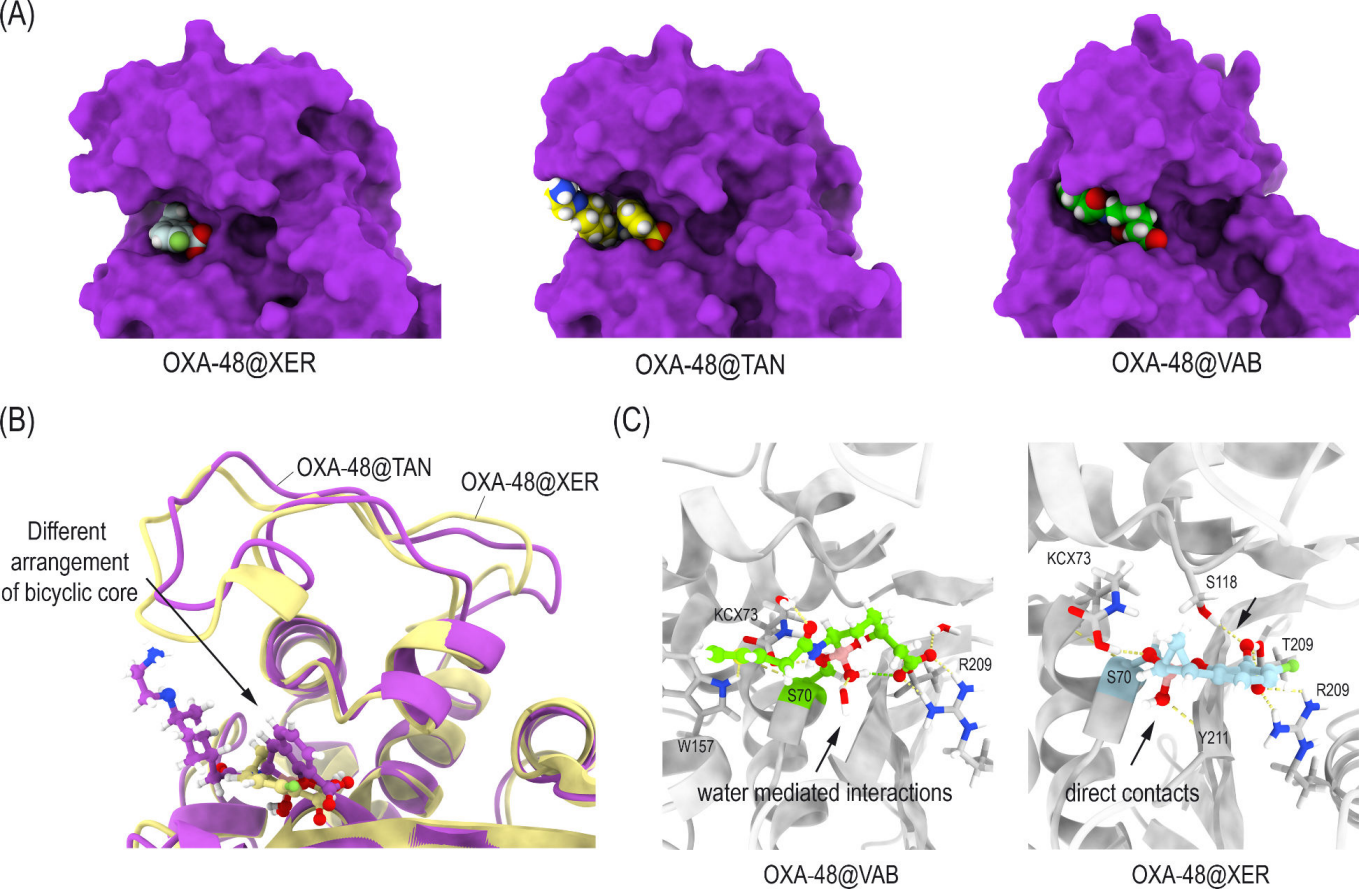
(**A**) Overall view of OXA-48 inactivated by bicyclic (XER and TAN) and cyclic (VAB) boronate inhibitors obtained by MD simulations. Snapshots taken after 90 ns of simulation. (**B**) Comparison of the binding conformation of modified TAN and XER in the OXA-48 active site. Note the distinct arrangement of the bicyclic core in both compounds. (**C**) Main contacts of the modified catalytic serine residue by VAB (green) and XER (blue). Relevant side chain residues are shown and labeled. Hydrogen-bonding interactions are shown as dashed yellow lines. Note how some strong direct interactions of modified XER are not observed in VAB.

In a previous study, we showed the inactivation of OXA-48 by xeruborbactam, taniborbactam, and vaborbactam in atomic detail showed important differences between the binding mode of the bicyclic (xeruborbactam and taniborbactam) and cyclic derivatives (vaborbactam) (16) (Fig. 3). By studying the binding of these boronate derivatives (Michaelis complex) in their sp2 form, we observed spontaneous deprotonation of the catalytic serine S70 by the carboxylated lysine residue K73 that triggered the subsequent nucleophilic attack of S70 to the boronate moiety (sp2 form), leading to formation of the enzyme adduct and a change to sp3 hybridization in the boron atom. For xeruborbactam, the modified inhibitor rotated ∼45° to adopt a horizontal arrangement on the active site allowing favorable CH–π and apolar interactions through both faces of the bicyclic moiety within the pocket. For taniborbactam, the insertion in the active site would be less effective, as the amido side chain, which is mainly exposed to the bulky solvent, adopts diverse conformations. This flexibility would also reduce the binding capacity for covalent modification, to some extent. On the other hand, the arrangement of the modified vaborbactam was quite different from those of xeruborbactam and taniborbactam. Thus, the equatorial arrangement of the amido group in vaborbactam and its less-restricted conformation favored the allocation of its side chain in the pocket formed by the Ω-loop in which W157 is located.

### Conclusions

In this study, we compared the activity of main BL/BLI combinations against the OXA-48 carbapenemase. Through biochemical studies, microbiological assays, and structural analyses, we identified the most effective BLIs for treating OXA-48-producing Enterobacterales. While combinations with DBOs, such as avibactam, zidebactam, nacubactam, and durlobactam appear to be attractive therapies due to their antimicrobial capacity and action as antibiotic enhancers, boronates, such as taniborbactam and xeruborbactam, are also very promising options as they are capable of significantly inhibiting this carbapenemase and the possible co-produced ESBLs.

Fortunately, we are now witnessing a rise in the development of new compounds with high therapeutic value against multidrug-resistant Gram-negative pathogens. These advancements offer new therapeutic options for previously difficult-to-treat infections, potentially reducing treatment costs and improving public health. Comparative studies are crucial for identifying the best treatments for challenging pathogens, such as OXA-48-producing Enterobacterales.

## SUPPLEMENTAL MATERIAL

Supplemental Material aac.01614-24-s0001.docx Supplemental methods, Tables S1 to S5, and Fig. S1 and S2.

ASM does not own the copyrights to Supplemental Material that may be linked to, or accessed through, an article. The authors have granted ASM a non-exclusive, world-wide license to publish the Supplemental Material files. Please contact the corresponding author directly for reuse.
