# Supplementary material for: Advancements in the fight against globally distributed OXA-48 carbapenemase: evaluating the new generation of carbapenemase inhibitors
Source: Antimicrob Agents Chemother. 2025 Jan 10;69(2):e01614-24. doi: 10.1128/aac.01614-24 (PMC11823609; doi:10.1128/aac.01614-24)
Supplement: Supplemental Material — Supplemental methods, Tables S1 to S5, and Fig. S1 and S2. [file aac.01614-24-s0001.docx]

**SUPPLEMENTARY MATERIAL AND METHODS**

**Advancements in the fight against worldwide-distributed OXA-48 carbapenemase: Evaluating the new generation of carbapenemase inhibitors.**

Michelle Outeda-García, Jorge Arca-Suárez, Emilio Lence, Arianna Rodriguez-Coello, Romina Maceiras, Tania Blanco-Martin, Paula Guijarro-Sánchez, Lucia Gonzalez-Pinto, Isaac Alonso-Garcia, Andrea García-Pose, Andrea Muras, Salud Rodriguez-Pallares, Cristina Lasarte-Monterrubio, Concepción González-Bello, Juan Carlos Vázquez-Ucha, German Bou, and Alejandro Beceiro.

Material and Methods. ………………………………………..Page 2

Supplementary Table 1. …………………………………….. Page 5

Supplementary Table 2. …………………………………….. Page 6

Supplementary Table 3. …………………………………….. Page 7

Supplementary Table 4. …………………………………….. Page 8

Supplementary Table 5. …………………………………….. Page 9

Supplementary Figure 1. ………………………….….…….. Page 10

Supplementary Figure 2. ……………………………..…….. Page 11

**Molecular cloning of *bla*_OXA-48_ gene**

To clone the *bla*_OXA-48_ gene in *E. coli* TG1 and *E. coli* HB4, the full-length gene was amplified by PCR from a clinical strain, digested with BamHI and EcoRI, ligated to the vector pUCP24 and finally transformed in *E. coli* strains. Similarly, the *bla*_CTX-M-14_ gene *was* digested with BamHI and EcoRI*,* ligated to pBGS18 vector and transformed in *E. coli* HB4 strain. The resulting transformants were selected in Luria-Bertani (LB) medium plates containing 10 mg/L gentamicin (pUCP24) and 50 mg/L of kanamycin (pBGS18).(1) For comparative purposes, empty plasmids (without *bla*_OXA-48_ and *bla*_CTX-M-14_ genes) were also transformed in *E. coli* strains.

**Purification of OXA-48 for Steady-State Kinetics**

For kinetic studies, the *bla*_OXA-48_ gene was cloned into the p-GEX-6p-1 plasmid to facilitate expression and purification (GE Healthcare, UK), as previously described (2). The gene was amplified from genomic DNA using a primer pair that included recognition sites for the restriction enzymes *Bam*HI (5′-AAAGGATCCAAGGAATGGCAAGAAAACAAA-3′) and *Eco*RI (5′-AAAGAATTCCTAGGGAATAATTTTTTCCTGTTT-3′). Later, the DNA fragment was digested with *Bam*HI and *Eco*RI and subsequently ligated into the *Bam*HI/*Eco*RI-digested p-GEX-6p-1 vector. These primers amplified the *bla*_OXA-48_ gene without the signal peptide, to prevent the fusion protein from migrating to the periplasm and thus facilitate its purification from the bacterial cytoplasm. The resulting recombinant plasmid was electroporated into a protease-deficient strain, *E. coli* BL21, with the aim of producing the fusion protein glutathione S-transferase (GST)/OXA-48. This protein was purified to homogeneity using the GST Gene Fusion System ([GE Healthcare](https://www.google.com/search?sa=X&sca_esv=599760920&biw=1920&bih=919&sxsrf=ACQVn088OflTFwOI3_YzISUc-SNV2aRl3g:1705662794784&q=GE+Healthcare&si=AKbGX_oBDfquzodaRrfbb9img4kPQ4fCBZjeqAiaW1svvC8uXqVg9ttjDQ97ptYJIaGEcSSgRltQTq85RRxXFM6NNYmwD1sQW85czWlADdrhtXwhcFv7VdEw6btcTtJRvTFrbYlrz8YvanVFqm_qtj0wzYeADFAd6ZsIo4_-MLbUVquTvu6is6mU1Asln0YZx5TxTUtxVipDvxStASFiE8aj-gyWa2ueLGDYBHKIqM7ek6j9pFJMJIRhsUCHnmlPKFUxXLEkS-SRoEJRW7pYOEmAUZsjOdbEVw%3D%3D&ved=2ahUKEwinlfWHqemDAxU7fKQEHRM9DlIQmxMoAXoECFQQAw), Il, USA), following the manufacturer's instructions. Purification was verified through SDS–PAGE gels, showing a band at approximately 29 kDa (≥95% purity). Further validation was performed using a MALDI-TOF/TOF spectrometer (Bruker Daltonics, MA, USA).

**Inhibition Kinetics. Affinity Binding, Acylation and Dissociation Assays.**

Inactivation of OXA-48 by novel BLIs was assessed with nitrocefin (MedChemExpress, NJ, USA) as a reporter substrate ([NFC] > 5x*K*_m NCF_). All experiments were conducted at 25 °C in phosphate-buffered saline (adding 20 mM NaHCO_3_ and 50 mM Na_2_HPO_4_), pH of 7.4 and monitored at λ=490 nm using an Epoch 2 Microplate spectrophotometer (Biotek, VE, USA). The results shown represent the mean values obtained from triplicate samples. The Michaelis constant (*K*_m_) for nitrocefin was determined following established protocols (3).

The apparent inhibition constant (*K_i_* _app_) is specific constant for competitive inhibition in the presence of nitrocefin. *K_i_* _app_ serves as an equilibrium constant, calculable from pre-steady-state conditions and indicates the formation of the enzyme acyl E-S complex. Concentrations of each inhibitor were systematically increased in a constant concentration of enzyme and nitrocefin mixtures, and the resulting initial velocities (*V*_0_) were recorded. Plotting the inverse initial velocities (1/*V*_0_) against inhibitory concentrations ([I]) facilitated fitting the data to a linear equation. The intersection, divided by the slope of the line, was defined as the observed *K_i_* _app_. The initial velocity (*V*_0_) was computed using equation 1, while the observed *K_i_* _app_ was fine-tuned for the presence of nitrocefin through equation 2 (4). Finally, *k_on_* (*k_2_/K*, the apparent onset of acylation) was calculated as the slope of the plot of [I] vs *k*_obs_ (5), and then corrected for nitrocefin affinity by using equation 3.

*V*_0_= (*V*_max_ × [S] /*K*_mNFC_ ) × (1+[I] /*K_i_* _app_) + [S] (1)

*K_i_* _app_ (corrected)= *K_i_* _app_ (observed) / [ 1+ ([S]/*K*_mNFC_)] (2)

*k*_2_/*K* = *k*_2_/*k*_obs_ × [([*S*]/*K*_m_ _NCF_) + 1] (3)

The turnover numbers (*t_n_*) or partition ratios, characterized by the inhibitor/enzyme ratio (I/E) required to achieve >90% inhibition of nitrocefin hydrolysis, were assessed after incubation for 15 min. The evaluation involved exposure to increasing concentrations of inhibitors plus 500 nM of enzyme, at different molar I/E ratios.

The dissociation rate constant (*k*_off_) was determined using a “jump dilution” method in which OXA-48 enzyme activity was monitored over time as the BLI dissociates. A concentration of 1 μM of OXA-48 was incubated with an excess of inhibitors (10x *K*_i app_), for 30 minutes at 37 °C. The incubation period was intentionally extended to ensure 100% inhibition. Subsequently, the reaction mixture was diluted 10,000-fold dilution in the reaction buffer. The assay was conducted in a 96-well microtiter plate, in which 197 μL of the diluted enzyme was combined with 3 µL of nitrocefin at 10 mM in 50 mM sodium phosphate and 20 mM sodium bicarbonate. OXA-48 activity was determined by monitoring the increase in absorbance in the UV-Vis spectrum due to nitrocefin hydrolysis using aliquots from the incubation samples and the control. Absorbance was recorded at 60-s intervals over a 2-h period at RT. The resulting reaction progress curve was fitted to equation 4, which includes the exponential function (6). *V*_0_ is the initial, completely inhibited enzyme velocity; *V*_s_ is the steady-state uninhibited enzyme velocity; and *t* is time. From the fit, GraphPad Prism software, (MA, USA) also calculates the dissociation half-time (*t*_1/2_), in min.

*k*_off_ was obtained directly from the dissociation experiment and *k_on_* was previously calculated, thus finally *K*_d_ was calculated as the fraction between *k*_off_ and *k*_on_**.**

P= V_s_ x *t* + (*V*_0_-*V*_s_) x [1– exp ^(-^*^k^*^off x^ *^t^*^)^] / *k*_off_  (4)

**Porin sequence analysis**

To identify truncated porins, a Python script was developed to automate the search and analysis process. First, reference sequences of specific porins for each species of interest were downloaded from the NCBI protein database. The analyzed porins included: *E. cloacae* complex (OmpC, OmpF, OmpA), *E. coli* (OmpC, OmpF, OmpN, OmpA) and *K. pneumoniae* (OmpK36, OmpK35, OmpK37, OmpA). The script translated the genomes to be analyzed in all six reading frames and performed BLAST searches of the reference sequences against these predicted proteomes. Based on homologies, the porins were grouped into OmpC/OmpK36/OmpE36, OmpF/OmpK35/OmpE35, OmpN/OmpK37, and OmpA. This result was classified as potentially truncated or non-functional if it had less than 90% coverage, less than 70% identity, or the presence of premature stop codons. Porins identified as potentially truncated or non-functional were subsequently subjected to manual review to confirm their status.

**Supplementary Table 1.** Description of the bacterial strains and transformants used in the study.

| **Strain** | **Description** | **β-lactams resistance genotype** | **Source or reference** |
| --- | --- | --- | --- |
| *E. coli* TG1 | *E. coli* reference strain, derived from *E. coli* K-12 | - | ATCC |
| *E. coli* TG1 + pUCP24/OXA-48 | Isogenic derivative of *E. coli* TG1 producing the carbapenemase OXA-48 | *bla*_OXA-48_ | (7) |
| *E. coli* HB4 | *E. coli* strain, derived from a *E. coli* clinical isolate | Δ*ompC*, Δ*ompF* | (8) |
| *E. coli* HB4 + pUCP24/OXA-48 | Isogenic derivative of *E. coli* HB4 producing the carbapenemase OXA-48 | Δ*ompC*, Δ*ompF, bla*_OXA-48_ | (7) |
| *E. coli* HB4 + pUCP24/OXA-48 + pBGS18/CTX-M-14 | Isogenic derivative of *E. coli* HB4 producing the carbapenemase OXA-48 and the ESBL CTX-M-14 | Δ*ompC*, Δ*ompF, bla*_OXA-48,_ *bla*_CTX-M-14_ | This study |

**Supplementary Table 2.** Bacterial species of OXA-48-producing clinical isolates, sequence types, and other produced β-lactamases.

| **Genome number** | **Strain number** | **Species** | **MLST*** | **ESBLs** | **Non-ESBL** | **Hospital (City)** |
| --- | --- | --- | --- | --- | --- | --- |
| AI2899 | 177 | *E. coli* | 131 | CTX-M-15 | OXA-1, AmpC | Ramón y Cajal University Hospital (Madrid) |
| AI2949 | 264 | *E. coli* | 131 | - | OXA-1, AmpC | Asturias University Hospital (Oviedo) |
| AI2853 | 103 | *E. coli* | 10 | CTX-M-14 | AmpC | Mutua de Terrassa University Hospital (Terrassa) |
| AI2938 | 224 | *E. coli* | 127 | - | AmpC | Son de Espases University Hospital (Palma de Mallorca) |
| AI2688 | 272 | *E. cloacae* | 78 | CTX-M-9 | ACT-24, | Asturias University Hospital (Oviedo) |
| AI2695 | 281 | *E. cloacae* | 78 | - | ACT-24 | Asturias University Hospital (Oviedo) |
| AI2662 | 235 | *E. cloacae* | 171 | CTX-M-9, CTX-M-15 | ACT-like, OXA-1 | Asturias University Hospital (Oviedo) |
| AH0328 | 231 | *E. cloacae* | 171 | - | ACT-like, OXA-1 | Son de Espases University Hospital (Palma de Mallorca) |
| AI2597 | 19 | *K. pneumoniae* | 11 | CTX-M-15 | SHV-11, OXA-1 | Gregorio Marañón University Hospital (Madrid) |
| AN2362 | 497 | *K. pneumoniae* | 11 | - | SHV-11, OXA-1 | Arquitecto Marcide Hospital (Ferrol) |
| AI2665 | 238 | *K. pneumoniae* | 15 | CTX-M-15, SHV-28 | OXA-1, TEM-1 | Asturias University Hospital (Oviedo) |
| AI2630 | 71 | *K. pneumoniae* | 15 | - | SHV-1 | Puerta del Mar Hospital (Cádiz) |
| AI2998 | 413 | *K. pneumoniae* | 147 | CTX-M-15 | SHV-11 | Bellvitge University Hospital (Barcelona) |
| AI2664 | 237 | *K. pneumoniae* | 147 | - | SHV-11, TEM-1, TEM-like | Asturias University Hospital (Oviedo) |
| AI2822 | 23 | *K. pneumoniae* | 392 | CTX-M-15 | SHV-11, OXA-1, TEM-1 | Gregorio Marañón University Hospital (Madrid) |
| AH0329 | 194 | *K. pneumoniae* | 392 | - | SHV-11 | La princesa University Hospital (Madrid) |
| AI2868 | 125 | *K. pneumoniae* | 307 | CTX-M-15 SHV-28 | OXA-1, TEM-1 | La Paz University Hospital (Madrid) |
| AI2896 | 170 | *K. pneumoniae* | 307 | - | SHV-1 | Ramón y Cajal University Hospital (Madrid) |

* Strains were chosen as part of the same sequence type (ST) in pairs (with/without ESBLs); except for one pair of *E. coli*, for which it was not possible to obtain a couple with/without ESBLs from the same ST, thus the genetically closer available isolate was selected.

**Table Supplementary 3.** Minimum inhibitory concentrations (mg/L) of ceftazidime in combination with novel β-lactamase inhibitors (4 mg/L) against *E. coli* TG1 and HB4 (low permeability) expressing OXA-48 carbapenemase and CTX-M-14 ESBL.

| **Strain** | **CAZ**^a^ | **CAZ/AVI** | **CAZ/REL** | **CAZ/ZID** | **CAZ/NAC** | **CAZ/DUR** | **CAZ/VAB** | **CAZ/TAN** | **CAZ/XER** |
| --- | --- | --- | --- | --- | --- | --- | --- | --- | --- |
| *E. coli* TG1 | 0.12 | ≤0.06* | 0.12 | ≤0.06* | ≤0.06* | ≤0.06* | 0.12 | 0.12 | 0.12 |
| *E. coli* TG1 (OXA-48) | 0.25 | ≤0.06* | 0.12 | ≤0.06* | ≤0.06* | ≤0.06* | 0.25 | 0.12 | 0.25 |
| *E. coli* HB4 Δ*ompC/F* | 1 | 0.5 | 1 | ≤0.06* | ≤0.06* | ≤0.06* | 1 | 1 | 1 |
| *E. coli* HB4 Δ*ompC/F* (OXA-48) | 1 | 0.5 | 1 | ≤0.06* | ≤0.06* | ≤0.06* | 1 | 1 | 1 |
| *E. coli* HB4 Δ*ompC/F* (CTX-M-14) | 16 | 0.5 | 2 | ≤0.06* | ≤0.06* | ≤0.06* | 2 | 1 | 1 |
| *E. coli* HB4 Δ*ompC/F* (CTX-M-14 + OXA-48) | 16 | 0.5 | 2 | ≤0.06* | ≤0.06* | ≤0.06* | 2 | 1 | 1 |

* No growth, the MICs to avibactam, zidebactam, nacubactam and durlobactam are lower than the used concentration (4 mg/L). ^a^ CAZ: ceftazidime; AVI: avibactam; REL: relebactam; ZID: zidebactam; NAC: nacubactam; DUR: durlobactam; VAB: vaborbactam; TAN: taniborbactam; XER: xeruborbactam

**Supplementary Table 4.** Minimum inhibitory concentrations (mg/L) of β-lactamase inhibitors alone against clinical Enterobacterales isolates.

| **Species** | | **Strain number** | **MLST** | **AVI**^a^ | **REL** | **ZID** | **NAC** | **DUR** | **VAB** | **TAN** | **XER** |
| --- | --- | --- | --- | --- | --- | --- | --- | --- | --- | --- | --- |
| *E. coli* | ESBL | 177 | 131 | 32 | ≥128 | 0.5 | 8 | 2 | ≥128 | ≥128 | 64 |
|  |  | 103 | 10 | 32 | ≥128 | 8 | 2 | 0.25 | ≥128 | ≥128 | 64 |
|  | No ESBL | 264 | 131 | 16 | ≥128 | 0.12 | 1 | 0.25 | ≥128 | ≥128 | 32 |
|  |  | 224 | 127 | 16 | ≥128 | 0.25 | 8 | 0.25 | ≥128 | ≥128 | 32 |
| *E. cloacae* | ESBL | 272 | 78 | 32 | ≥128 | 0.5 | 4 | 1 | ≥128 | ≥128 | 32 |
|  |  | 235 | 171 | 32 | ≥128 | 2 | 8 | 4 | ≥128 | ≥128 | ≥128 |
|  | No ESBL | 281 | 78 | 16 | ≥128 | 0.25 | 4 | 4 | ≥128 | ≥128 | 32 |
|  |  | 231 | 171 | 8 | ≥128 | 0.25 | 1 | 1 | ≥128 | ≥128 | 32 |
| *K. pneumoniae* | ESBL | 19 | 11 | ≥128 | ≥128 | ≥128 | ≥128 | 2 | ≥128 | ≥128 | 32 |
|  |  | 238 | 15 | 32 | ≥128 | 0.25 | 16 | 0.5 | ≥128 | ≥128 | 64 |
|  |  | 413 | 147 | 64 | ≥128 | ≥128 | ≥128 | 4 | ≥128 | ≥128 | 64 |
|  |  | 23 | 392 | 16 | ≥128 | 0.5 | 2 | 0.5 | ≥128 | ≥128 | 32 |
|  |  | 125 | 307 | 32 | ≥128 | ≥128 | ≥128 | 2 | ≥128 | ≥128 | 32 |
|  | No ESBL | 497 | 11 | ≥128 | ≥128 | 32 | ≥128 | 2 | ≥128 | ≥128 | 64 |
|  |  | 71 | 15 | 32 | ≥128 | 0.5 | 16 | 2 | ≥128 | ≥128 | 32 |
|  |  | 237 | 147 | 64 | ≥128 | 0.5 | 1 | 0.5 | ≥128 | ≥128 | 64 |
|  |  | 194 | 392 | 16 | ≥128 | 0.5 | 2 | 1 | ≥128 | ≥128 | 32 |
|  |  | 170 | 307 | 16 | ≥128 | 0.5 | 4 | 0.5 | ≥128 | ≥128 | 32 |

^a^ AVI: avibactam; REL: relebactam ; ZID: zidebactam; NAC: nacubactam; DUR: durlobactam; VAB: vaborbactam; TAN: taniborbactam; XER: xeruborbactam.

**Supplementary Table 5.** Minimum inhibitory concentrations (mg/L) **of** ceftazidime in combination with novel β-lactamase inhibitors (4 mg/L) against Enterobacterales clinical isolates carrying OXA-48, and with ESBLs (n=9) or without ESBLs (n=9).

| **Species** | | **Strain number** | **MLST** | **CAZ**^a^ | **CAZ/AVI** | **CAZ/REL** | **CAZ/ZID** | **CAZ/NAC** | **CAZ/DUR** | **CAZ/VAB** | **CAZ/TAN** | **CAZ/XER** |
| --- | --- | --- | --- | --- | --- | --- | --- | --- | --- | --- | --- | --- |
| *E. coli* | ESBL | 177 | 131 | 128 | 0.25 | 1 | ≤0.06* | 0.25 | ≤0.06* | 4 | 0.25 | 0.5 |
|  |  | 103 | 10 | 4 | 0.5 | 0.5 | ≤0.06 | ≤0.06* | ≤0.06* | 0.5 | 0.5 | 0.25 |
|  | No ESBL | 264 | 131 | 0.25 | 0.25 | 0.25 | ≤0.06* | ≤0.06* | ≤0.06* | 0.25 | 0.25 | 0.25 |
|  |  | 224 | 127 | 0.25 | 0.25 | 0.12 | ≤0.06* | ≤0.06* | ≤0.06* | 0.25 | 0.25 | 0.25 |
| *E. cloacae* | ESBL | 272 | 78 | ≥256 | 1 | 0.5 | ≤0.06* | ≤0.06* | ≤0.06* | 16 | 2 | 8 |
|  |  | 235 | 171 | 128 | 1 | 2 | ≤0.06* | 0.25 | ≤0.06 | 16 | 1 | 2 |
|  | No ESBL | 281 | 78 | 0.5 | 0.25 | 0.5 | ≤0.06* | ≤0.06* | ≤0.06* | 0.5 | 0.25 | 0.25 |
|  |  | 231 | 171 | 1 | 0.12 | 0.5 | ≤0.06* | ≤0.06* | ≤0.06* | 1 | 0.5 | 0.5 |
| *K. pneumoniae* | ESBL | 19 | 11 | 32 | 0.5 | 0.5 | 0.12 | 0.12 | ≤0.06* | 2 | 0.5 | 0.5 |
|  |  | 238 | 15 | 32 | 0.5 | 0.5 | ≤0.06* | ≤0.06 | ≤0.06* | 2 | 0.5 | 1 |
|  |  | 413 | 147 | 32 | 0.5 | 0.5 | ≤0.06 | ≤0.06 | ≤0.06 | 4 | 0.5 | 0.5 |
|  |  | 23 | 392 | 128 | 0.5 | 1 | ≤0.06* | ≤0.06* | ≤0.06* | 2 | 0.5 | 0.5 |
|  |  | 125 | 307 | 64 | 0.5 | 0.5 | ≤0.06 | ≤0.06 | ≤0.06* | 2 | 0.5 | 1 |
|  | No ESBL | 497 | 11 | 0.5 | 0.25 | 0.25 | ≤0.06 | ≤0.06 | ≤0.06* | 0.5 | 0.5 | 0.25 |
|  |  | 71 | 15 | 0.5 | 0.5 | 0.5 | ≤0.06* | ≤0.06 | ≤0.06* | 0.5 | 0.5 | 0.5 |
|  |  | 237 | 147 | 0.25 | 0.25 | 0.25 | ≤0.06* | ≤0.06* | ≤0.06* | 0.25 | 0.25 | 0.25 |
|  |  | 194 | 392 | 0.5 | 0.5 | 0.5 | ≤0.06* | ≤0.06* | ≤0.06* | 0.5 | 0.5 | 0.5 |
|  |  | 170 | 307 | 0.5 | 0.25 | 0.25 | ≤0.06* | ≤0.06* | ≤0.06* | 0.5 | 0.12 | 0.25 |

* No growth, the MICs to zidebactam, nacubactam and durlobactam are lower than the used concentration (4 mg/L). CAZ^a^: ceftazidime; AVI: avibactam; REL: relebactam; ZID: zidebactam; NAC: nacubactam DUR: durlobactam; VAB: vaborbactam; TAN: taniborbactam; XER: xeruborbactam.

**
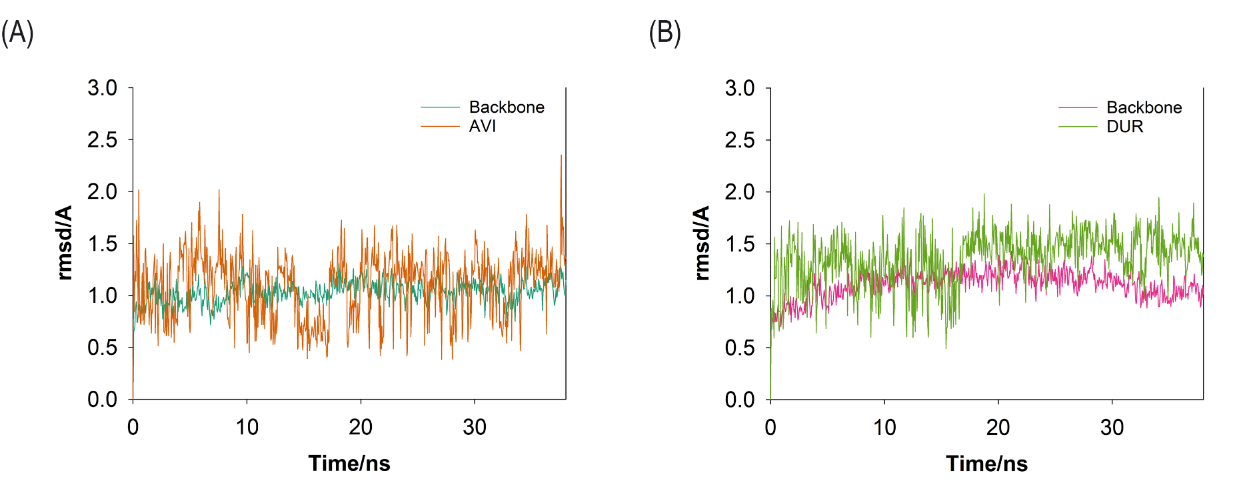
**

**Supplementary Figure 1.** RMSD plots for the OXA-48 backbone (Cα, C, O and N atoms) and β-lactamase inhibitors, avibactam and durlobactam, from the MD simulations of the OXA-48@avibactam (A) and OXA-48@durlobactam (B) complexes, respectively. Note how both the protein and the ligand were stable in the active site throughout the simulation as no significant changes were observed.

**
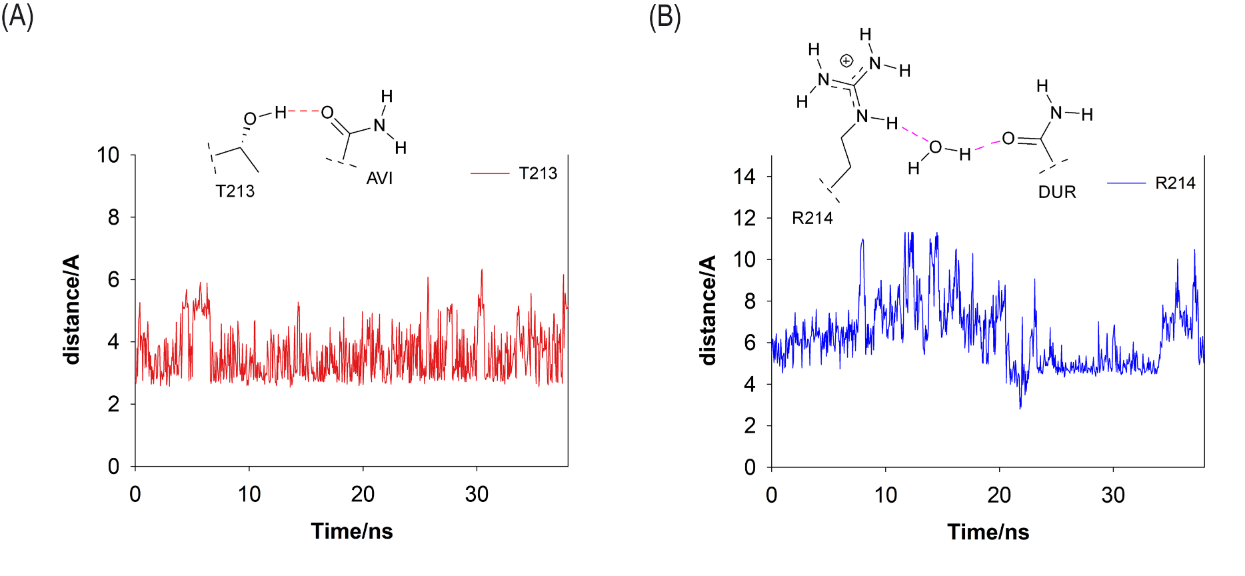
**

**Supplementary Figure 2.** Stability of the hydrogen-bonding interactions of the amido moiety in avibactam (A) with T213 and durlobactam (B) with R214 calculated from the MD simulations of the OXA-48@avibactam and OXA-48@durlobactam Michaelis complexes, respectively. Variation of the relative distances between (A) the carbonyl group (O9 atom) in AVI and the hydroxyl group in T213 (OG1 atom) and (B) the carbonyl group (O9 atom) in DUR and the guanidinium group (NE atom) in R214. Note that the interactions were measured between heavy atoms (O atoms) and the water molecule in (B) was not considered due to the exchange in water.

**REFERENCES**

1. Spratt BG, Hedge PJ, te Heesen S, Edelman A, Broome-Smith JK. 1986. Kanamycin-resistant vectors that are analogues of plasmids pUC8, pUC9, pEMBL8 and pEMBL9. Gene 41:337–342.

2. Vallejo JA, Martínez-Guitián M, Vázquez-Ucha JC, González-Bello C, Poza M, Buynak JD, Bethel CR, Bonomo RA, Bou G, Beceiro A. 2016. LN-1-255, a penicillanic acid sulfone able to inhibit the class D carbapenemase OXA-48. J Antimicrob Chemother 71:2171–2180.

3. Papp-Wallace KM, Mallo S, Bethel CR, Taracila MA, Hujer AM, Fernández A, Gatta JA, Smith KM, Xu Y, Page MGP, Desarbre E, Bou G, Bonomo RA. 2014. A kinetic analysis of the inhibition of FOX-4 β-lactamase, a plasmid-mediated AmpC cephalosporinase, by monocyclic β-lactams and carbapenems. J Antimicrob Chemother 69:682–690.

4. Winkler ML, Papp-Wallace KM, Hujer AM, Domitrovic TN, Hujer KM, Hurless KN, Tuohy M, Hall G, Bonomo RA. 2015. Unexpected challenges in treating multidrug-resistant Gram-negative bacteria: resistance to ceftazidime-avibactam in archived isolates of Pseudomonas aeruginosa. Antimicrob Agents Chemother 59:1020–1029.

5. Papp-Wallace KM, Winkler ML, Taracila MA, Bonomo RA. 2015. Variants of β-lactamase KPC-2 that are resistant to inhibition by avibactam. Antimicrob Agents Chemother 59:3710–3717.

6. R T, O L. 2020. Biochemical Activity of Vaborbactam. Antimicrobial agents and chemotherapy 64.

7. Alonso-García I, Vázquez-Ucha JC, Martínez-Guitián M, Lasarte-Monterrubio C, Rodríguez-Pallares S, Camacho-Zamora P, Rumbo-Feal S, Aja-Macaya P, González-Pinto L, Outeda-García M, Maceiras R, Guijarro-Sánchez P, Muíño-Andrade MJ, Fernández-González A, Oviaño M, González-Bello C, Arca-Suárez J, Beceiro A, Bou G. 2023. Interplay between OXA-10 β-Lactamase Production and Low Outer-Membrane Permeability in Carbapenem Resistance in Enterobacterales. Antibiotics (Basel) 12:999.

8. Mammeri H, Nordmann P, Berkani A, Eb F. 2008. Contribution of extended-spectrum AmpC (ESAC) beta-lactamases to carbapenem resistance in Escherichia coli. FEMS Microbiol Lett 282:238–240.
